# By-Product Extracts from *Castanea sativa* Counteract Hallmarks of Neuroinflammation in a Microglial Model

**DOI:** 10.3390/antiox12040808

**Published:** 2023-03-26

**Authors:** Pasquale Marrazzo, Manuela Mandrone, Ilaria Chiocchio, Laura Zambonin, Maria Cristina Barbalace, Chiara Zalambani, Cristina Angeloni, Marco Malaguti, Cecilia Prata, Ferruccio Poli, Diana Fiorentini, Silvana Hrelia

**Affiliations:** 1 Department of Experimental, Diagnostic and Specialty Medicine, Alma Mater Studiorum—University of Bologna, Via Belmeloro, 8, 40126 Bologna, Italy; 2 Department of Pharmacy and Biotechnology, Alma Mater Studiorum—University of Bologna, Via Irnerio, 42, 40126 Bologna, Italy; 3 Department of Pharmacy and Biotechnology, Alma Mater Studiorum—University of Bologna, Via Irnerio, 48, 40126 Bologna, Italy; 4 Department for Life Quality Studies, Alma Mater Studiorum—University of Bologna, Corso D’Augusto 237, 47921 Rimini, Italy

**Keywords:** *Castanea sativa*, chestnut by-products, microglia, neuroinflammation, BV-2, flavonoids, waste valorisation, Toll-like receptor 4, NF-kB

## Abstract

*Castanea sativa* is very common in Italy, and the large amount of waste material generated during chestnut processing has a high environmental impact. Several studies demonstrated that chestnut by-products are a good source of bioactive compounds, mainly endowed with antioxidant properties. This study further investigates the anti-neuroinflammatory effect of chestnut leaf and spiny bur extracts, together with the deepest phytochemical characterisation (by NMR and MS) of active biomolecules contained in leaf extracts, which resulted in being more effective than spiny bur ones. BV-2 microglial cells stimulated with lipopolysaccharide (LPS) were used as a model of neuroinflammation. In BV-2 cells pre-treated with chestnut extracts, LPS signalling is partially blocked via the reduced expression of TLR4 and CD14 as well as the expression of LPS-induced inflammatory markers. Leaf extract fractions revealed the presence of specific flavonoids, such as isorhamnetin glucoside, astragalin, myricitrin, kaempferol 3-rhamnosyl (1-6)(2″-trans-p-coumaroyl)hexoside, tiliroside and unsaturated fatty acids, all of which could be responsible for the observed anti-neuroinflammatory effects. Interestingly, the kaempferol derivative has been identified in chestnut for the first time. In conclusion, the exploitation of chestnut by-products is suitable for the achievement of two goals: satisfaction of consumers’ demand for new, natural bio-active compounds and valorisation of by-products.

## 1. Introduction

Health and environmental care have recently attracted considerable interest among modern consumers. In this regard, the increasing demand for natural and safe bioactive products has triggered the research of new sources of natural active molecules. The undervalued by-products derived from forestry and agriculture can be considered an inexpensive source of such compounds. The exploitation of agro-forestry residues can lead to a double benefit: (i) the disposal of large quantities of waste, representing a source of pollution; (ii) the production of valuable products.

*Castanea sativa* by-product extracts are rich in many phenolic compounds endowed with antioxidant activity, as reported in recent studies performed in different cell models and in vitro [1,2,3,4].

The main compounds found in the waste of *Castanea sativa* are phenolic acids, flavonoids and hydrolysable tannins [5,6], responsible for the antioxidant [7], anticancer [8,9,10], and anti-inflammatory [11,12] effects observed in several cell models.

*Castanea sativa* Mill. (Fagaceae), commonly called chestnut, is widespread in the Mediterranean area, and Italy is the largest chestnut producer in Europe, supporting the industrial production of marron glace, purees and chestnut flour. The chestnut harvesting and industrial processes produce solid by-products, constituted by inner and outer shells, leaves and spiny burs, whose removal represents an environmental problem, especially when shells and burs are burned. In this scenario, waste valorisation and re-use strategies appear to be more sustainable solutions, with the aim of establishing circular economy practices.

In this regard, we recently evaluated the anti-inflammatory and protective effects of leaf and spiny bur extracts from different *C. sativa* cultivar in BV-2 microglia cells exposed to lipopolysaccharide (LPS) as a cell model of neuroinflammation [13]. Microglia cells exert the immune surveillance of the central nervous system, playing a role in host defence and tissue repair in the brain. Several studies have evidenced a strong association between microglia-mediated neuroinflammation and the pathogenesis of many neurological and neurodegenerative diseases, such as Huntington’s, Parkinson’s and Alzheimer’s diseases [14,15,16,17]. One of the main features of neuroinflammation is the activation of microglia, which reacts to abnormal stimulations, such as pathogenic endotoxins, including LPS, located in the outer membrane of Gram-negative bacteria, neurotoxins or injury [18]. Excessive activation of microglial cells can be induced through the binding of LPS to the Toll-like receptor 4 (TLR4). TLR4 binds LPS with the help of the plasma membrane-anchored CD14 [19], which also controls the consequent internalisation of the LPS-activated TLR4, crucial for receptor signalling and degradation [20].

TLR4, together with the help of CD14, triggers numerous downstream signal transduction pathways, including the one mediated by nuclear factor NF-kB, that lead to the production of a series of pro-inflammatory agents, which in turn cause neuronal dysfunction and cell death [21]. LPS-induced overactivation of microglial cells can also lead to oxidative stress by inducing the release of reactive oxygen species (ROS), which further exacerbates the inflammatory pattern. Thus, the inhibition of the excessive activation of microglia is a relevant strategy to counteract the onset and progression of several brain diseases [22].

Our previous results showed that upon LPS-induced activation, BV-2 cells pre-treated with different *C. sativa* extracts showed a significant reduction of the transcriptional level of IL-1β and TNF-α and a diminished amount of NF-kB [13].

Starting from these results, the present study aims to deeply investigate the effects of leaf and spiny bur extracts in LPS-activated BV-2 cells, focusing on their anti-inflammatory molecular mechanisms and trying to associate the observed effect to specific classes of compounds.

## 2. Materials and Methods

### 2.1. Sampling and Extracts Preparation

*Castanea sativa* spiny burs and leaves were collected from 25 different trees (including different cultivars of both “Castagna” and “Marrone”) [13] in the experimental grove of Granaglione (Bologna, Italy, 44°16′ N, 10°90′ E). The collected material was dried and powdered, as reported by Chiocchio et al. [13]. The collected material was pooled in order to obtain one representative sample for leaves and one for spiny burs.

One litre of MeOH/H_2_O (1:1) mixture was used to extract 80 g of leaves or spiny burs. The extracts were sonicated for 30 min, then centrifuged for 20 min (2469× *g*), filtered on a Büchner funnel and dried at 40 °C by using a rotary evaporator (R215, Buchi, Flawil, Switzerland), obtaining dry leaf (L) and spiny bur (SB) extracts, which were tested in the bioassays.

### 2.2. Materials

Mini-PROTEAN^®^ TGX™ precast gels 4–20%, Precision Plus Protein™ Unstained Standards, Clarity™ Western ECL Substrate, and DC™ protein assay were bought from Bio-Rad Laboratories (Segrate, Milano, Italy). Ultra-low Endotoxin FBS was purchased from Euroclone (Pero, Milano, Italy). Rabbit anti-TLR4 polyclonal FITC-conjugated antibody was purchased from StressMarq. Rabbit monoclonal anti-NF-kB (#8242), rabbit monoclonal anti-p-NF-kB (#3031), rabbit monoclonal anti-NLRP3 (#15101), rabbit monoclonal anti-iNOS (#13120) or anti-rabbit IgG, HRP-linked (#7074) antibodies were purchased from Cell Signalling Technology (Danvers, MA, USA)**.** ELISA kit for TNF-α quantification and Griess (G4410) reagent for nitrite quantification was from Sigma-Aldrich (Saint Louis, MO, USA).

Deuterium oxide (D_2_O, 99.90% D) and CD_3_OD (99.80% D) were purchased from Eurisotop (Cambridge Isotope Laboratories, Inc., Saint-Aubin, France). Lipopolysaccharide (LPS) from *E. coli* O127:B8, mouse anti-tubulin antibody, FITC-conjugated LPS from *E. coli* O111:B4, standard 3-(trimethylsilyl)-propionic-2,2,3,3-d4 acid sodium salt (TMSP), sodium phosphate dibasic anhydrous, sodium phosphate monobasic anhydrous, phosphate-buffered saline (PBS), Dulbecco’s phosphate-buffered saline (DPBS), Accutase and all other solvents and chemicals were purchased from Sigma-Aldrich Co. (St. Louis, MO, USA).

### 2.3. Cell Culture and Treatments

BV-2 murine microglial cells were kindly provided by Prof. Elisabetta Blasi (the University of Modena and Reggio Emilia, Modena, Italy). BV-2 cells were cultured in DMEM with Ultra-low Endotoxin FBS 10% inactivated by heat treatment, L-glutamine 2 mM and penicillin-streptomycin solution in a humidified incubator at 5% CO_2_ and 37 °C.

Cells were seeded at a concentration of 3 × 10^4^ cells/cm^2^ in tissue culture plates, then treated for 3 h with spiny bur (SB) or leaf extracts (L) or with L sub-fractions obtained by liquid–liquid partition (0.5 mg/mL) here below described. All the dried fractions (Fr) were resuspended in a stock solution of DMSO, apart from Fr. H_2_O. As usual, the final DMSO concentration in the cell medium was <1% and no differences from the control cells were observed in all performed experiments. Subsequently, 0.5 μg/mL of LPS was added for a further 21 h. Unstimulated samples were used as a negative control of inflammation.

### 2.4. LPS Binding Assay

To evaluate the binding of LPS to BV-2 cells in the presence of the extracts, a fluorescent LPS was incubated with cultured cells, according to [23], with slight modifications. Cells were treated with extracts and then with FITC-LPS (0.5 μg/mL) in a complete medium. At 24 h, cells were detached with Accutase solution and pelleted in tubes. Cells were then resuspended in PBS, and the median fluorescence intensity (MFI) of the FITC-signal was analysed by flow cytometry. Flow data were collected using Guava^®^ easyCyte™ 5HT flow cytometer (Luminex Corporation, Austin, TX, USA) and processed via FlowJo software. A decrease in fluorescence correlates with an inhibition of the binding of LPS to BV-2 cells. Samples not incubated with labelled-LPS were used as controls.

### 2.5. Surface TLR4 Expression

The surface expression of TLR4 was evaluated as previously reported [24]. Briefly, 3 × 10^4^ cells/cm^2^ were initially seeded in tissue culture plates, treated with chestnut extracts and stimulated (or not) with LPS (0.5 μg/mL). The culture medium was removed, and the adherent cells were collected after incubation in Accutase solution, following centrifugation at 300× *g* for 5 min. The sample pellets were washed twice, centrifuged and resuspended in 0.2% BSA-DPBS. Cells were stained with anti-TLR4 FITC-conjugated antibody 1:100 (*v*/*v*) and incubated for 30 min at 37 °C according to the manufacturer’s instructions. After two washes in BSA-DPBS, cells were resuspended up to 5 × 10^5^ cells/mL in 0.1% BSA-PBS. Guava^®^ easyCyte™ 5HT flow cytometer was used to measure TLR4 FITC-fluorescence. FlowJo software was used to calculate the median fluorescence intensity (MFI). Unstained samples were used to measure the background signal.

### 2.6. Western Blot Analysis

The protein expression of NF-kB, p-NF-kB, iNOS, and NLRP3 was detected by Western blotting, as previously reported [13]. BV-2 cells were treated with the extracts (0.5 mg/mL) for 3 h and then co-treated with LPS (0.5 μg/mL) for a further 21 h. Cells were then lysed with RIPA buffer plus protease and phosphatase inhibitor cocktails. Bio-Rad DC protein assay was used to define the lysates protein concentration. Proteins (10 μg per lane) were separated on precast gels (Bio-Rad-Laboratories Inc.) by electrophoresis and transferred to a nitrocellulose membrane. After blocking, nitrocellulose membranes were incubated overnight with rabbit monoclonal anti-NF-kB, anti-p-NF-kB, anti-NLRP3, anti-iNOS or anti-actin antibodies in TBS-Tween containing 0.5% BSA at 4 °C. Secondary antibodies were incubated for 1 h at room temperature. Chemiluminescent bands were acquired by ChemiDoc (Bio-Rad) camera, and densitometry was calculated using Image Lab software (Bio-Rad) analysis.

### 2.7. TNF-α ELISA

In cell media, tumour necrosis factor-alpha (TNF-α) quantification was performed by enzyme-linked immunosorbent assay (ELISA), according to the manufacturer’s instructions (Merck KGaA, Darmstadt, Germany). For each sample, TNF-α concentration was calculated by interpolating the data with the linear equation of a calibration curve obtained with known cytokine concentrations.

### 2.8. Nitrite Quantification

To assess the production of nitric oxide (NO) from BV-2 cells, the extracellular release of nitrite (NO_2_^−^) was measured by Griess reagent according to the manufacturer’s instructions (Merck KGaA).

### 2.9. RT-PCR Analysis

Active gene expression was detected by tracking gene transcription. As previously reported, the level of mRNA expression in BV-2 cells was measured by reverse transcriptase polymerase chain reaction (RT-PCR) [25,26].

After treatment with chestnut extracts or fractions and stimulation with LPS, cells were lysed, and total RNA extraction was performed using the RNeasy Mini kit (Qiagen, Hilden, Germany). Total RNA quantification was evaluated through a NanoVue spectrophotometer, and mRNA reverse transcription was performed using an iScript cDNA synthesis kit (Bio-Rad). For real-time PCR, the SsoAdvanced SYBR Green mix (Bio-Rad), cDNA reverse transcribed from the samples and specific primer pairs (Table 1) were mixed according to the manufacturer’s instructions.

Normalised expression levels were calculated relative to control cells according to the 2^−ΔΔCT^ method.

### 2.10. Liquid/Liquid Partition and Medium Pressure Liquid Chromatography (MPLC) of Leaf Extract

Chestnut leaf extract was fractionated through liquid–liquid partition as described in our previous work [13]. In particular, the extract was suspended in 300 mL MeOH solution (250 mL H_2_O and 50 mL MeOH) and then extracted by liquid/liquid partition with 250 mL of hexane, ethyl acetate, and n-butanol (four times for each solvent). After this procedure, solvents were evaporated by a rotary evaporator, obtaining four fractions, respectively hexane (Fr. Hex 1.6 g), ethyl acetate (Fr. EtOAc 3.2 g), n-butanol (Fr. But 10.2 g) and water (Fr. H_2_O 21.2 g), which were tested in the bioassays and analysed by ^1^H NMR profiling.

Fr. EtOAc, which contained the bioactive flavonoids, was further purified by an MPLC instrument (Reveleris^®^, Büchi, Flawil, Switzerland) equipped with a UV-detector and fraction collector and by using a C18 column (4 g) and following the method performed in our previous work [13], leading to the obtaining of 13 sub-fractions (from FR1 to FR13). The fractions were analysed by mono- and two-dimensional NMR and MS showing the presence of myricetin (FR6), kaempferol (FR6), isorhamnetin glucoside (FR6), and a new flavonoid kaempferol 3-rhamnosyl (1-6)(2″-trans-p-coumaroyl)hexoside (FR8).

### 2.11. NMR and UHPLC-MS Measurement

^1^H NMR spectra, ^1^H-^1^H homonuclear (COSY) and inverse detected ^1^H-^13^C correlation experiments (HMBC, HSQC) were recorded at 25 °C on a Varian Inova instrument (equipped with a reverse triple resonance probe). CD_3_OD was used for an internal lock. For ^1^H, NMR profiling of the fractions obtained by liquid/liquid partition was prepared at a concentration of 10 mg/mL in CD_3_OD. Fr. H_2_O was prepared in CD_3_OD:D_2_O (50:50). The relaxation delay was 2.0 s, observed pulse 5.80 µs, number of scans 256, acquisition time 16 min, and spectral width 9595.78 Hz (corresponding to 16.0). For the aqueous samples, a pre-saturation sequence (PRESAT) was used to suppress the residual H_2_O signal at 4.83 (power = 6 dB, pre-saturation delay 2 s).

UHPLC-MS analyses were run on a Waters ACQUITY ARC UHPLC/MS system consisting of a QDa mass spectrometer equipped with an electrospray ionisation interface and a 2489 UV/Vis detector. The detected wavelengths were 210, 254 and 365 nm. The analyses were performed on an XBridge BEH C18 column (10 × 2.1 mm i.d., particle size 2.5 μm) with an XBridge BEH C18 VanGuard Cartridge precolumn (5 mm × 2.1 mm i.d., particle size 1.8 μm). The mobile phases were H_2_O (0.1% formic acid) (A) and MeCN (0.1% formic acid) (B). Electrospray ionisation in positive and negative modes was applied in the 50–1200 Da mass scan range.

### 2.12. Statistical Analysis

Each experiment was performed at least in triplicate. The column charts show the mean values of all performed experiments, with each bar representing a sample group that includes controls or treatments. The presented data show scores that were associated with standard error of measurement (SEM). To assess significant differences between the groups, a one-way ANOVA method with Fisher’s LSD correction was applied. Values were considered significantly different for *p*-value = 0.05 and specifically labelled by symbols as indicated in each figure legend. Statistical analysis was supported by GraphPad Prism software (Prism 9.5.0).

## 3. Results

### 3.1. Effect of Chestnut Extracts on the Interaction between LPS and TLR4 in BV-2 Microglial Cells

LPS triggers microglial cell activation by binding to its main cell receptor, TLR4, which is predominantly expressed in the brain’s microglia. TLR4 further activates different downstream signal transduction pathways, including the NF-kB signalling pathway, that in turn lead to the transcription of pro-inflammatory genes responsible for the onset of neuroinflammation and neurodegeneration [27].

In a previous work, we demonstrated the cytoprotective and anti-inflammatory action of chestnut leaf and spiny bur extracts derived from different *Castanea sativa* cultivars. To clarify the underlying mechanism of these observed effects, in this study, we first investigated whether spiny bur (SB) or leaf (L) hydroalcoholic extracts (obtained from pooling material collected from different trees) could somehow modulate the binding of LPS to TLR4.

**Figure 1:**
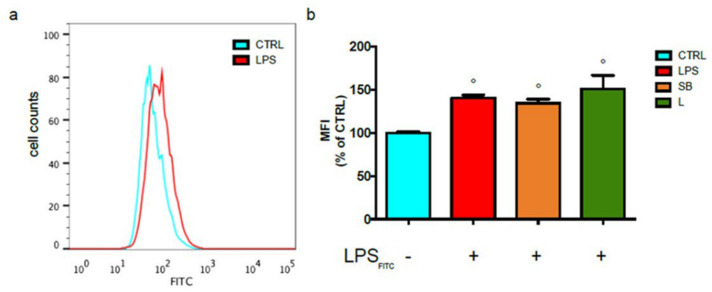
Analysis of LPS binding to BV-2 cells after treatment with chestnut leaf or spiny bur extracts. BV-2 cells were pre-treated (or not) with spiny bur (SB) or leaf (L) extracts (0.5 mg/mL) for 3 h, then incubated with 0.5 μg/mL LPS-FITC for a total of 24 h. The fluorescence of bound LPS was evaluated by flow cytometric analysis of live cells. (**a**) Representative plot of untreated controls and LPS-treated cells (**b**) Relative quantification is expressed as MFI, the median fluorescence intensity. Results are expressed as means ± SEM of three independent experiments. Statistical analysis was performed by Fisher’s LSD test following one-way ANOVA. ° *p* < 0.05 significantly different from control cells.

To this purpose, BV-2 cells treated with SB or L and stimulated with labelled LPS were analysed by flow cytometry (Figure 1).

Results reported in Figure 1a show that the fluorescence intensity is clearly increased in BV-2 cells stimulated with LPS compared to the unstimulated control. Of note, pre-treatment with chestnut extracts did not modulate fluorescence intensity, suggesting that the extracts do not influence the binding of LPS to TLR4 (Figure 1b).

### 3.2. Effect of Chestnut Extracts on the Level of TLR4 Exposed on the Surface of BV-2 Microglial Cells

**Figure 2:**
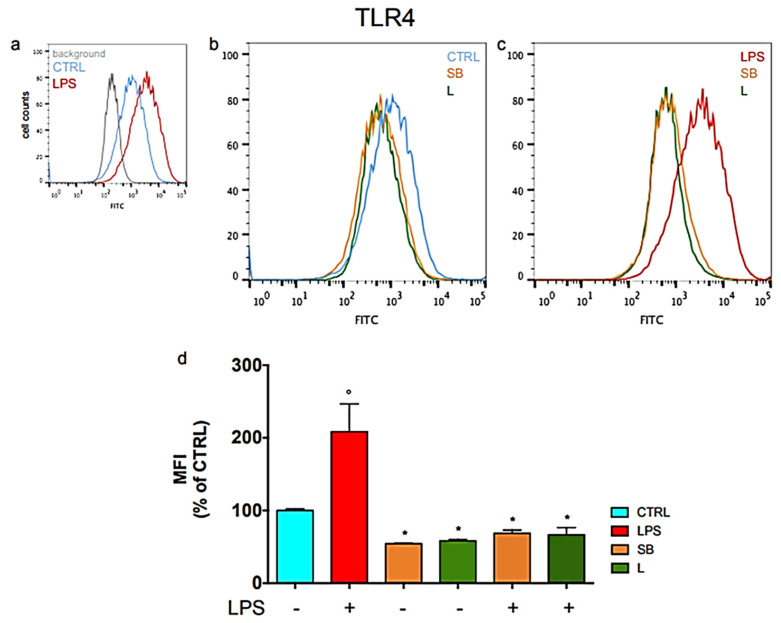
Cell surface level of TLR4 in BV-2 cells treated with chestnut leaf or spiny bur extracts and LPS. BV-2 cells were pre-treated (or not) with spiny bur (SB) or leaf (L) extracts (0.5 mg/mL) for 3 h, incubated with or without LPS (0.5 μg/mL) for a total of 24 h, then subjected to flow cytometric analysis of the immunostaining for TLR4 receptor. (**a**) Representative plots of untreated BV-2 cells (**b**) Representative plots of BV-2 cells not activated with LPS (**c**) Representative plots of LPS-stimulated BV-2 cells (**d**) Relative quantification is expressed as MFI, median fluorescence intensity. Results are expressed as means ± SEM of three independent experiments. Statistical analysis was performed by Fisher’s LSD test following one-way ANOVA. ° *p* < 0.05 significantly different from control cells; * *p* < 0.05 significantly different from LPS-treated cells.

Having ensured that the extracts do not influence the link between LPS and TLR4, we investigated whether the extracts modulate the expression of TLR4 on cell membranes. To this purpose, BV-2 cells were treated with the extracts, exposed to LPS and stained with anti-TLR4 fluorophore-conjugated antibody before acquiring the fluorescence by flow cytometry (Figure 2).

Figure 2a displays a clear peak shift in LPS-stimulated cells in comparison to controls, indicating a higher expression of TLR4 on the cell membrane upon LPS treatment. Figure 2c shows that treating LPS-stimulated BV-2 cells with SB or L causes a peak shift toward lower fluorescence values with respect to LPS-stimulated untreated cells, thus reducing the signal of TLR4. Interestingly, the fluorescence of TLR4 is also reduced in unstimulated BV-2 cells pre-treated with the chestnut extracts, as shown in Figure 2b. The relative quantification of these data, reported in Figure 2d, shows that the pre-treatment of BV-2 cells with SB and L leads to a fluorescence reduction of about 54–58% with respect to controls, which is also maintained when cells are stimulated with LPS. No significant difference is observed between the effects of the two extracts.

These results indicate that SB and L can reduce the presence of TLR4 on the surface of BV-2 cells, thus diminishing TLR4 signalling and, consequently, limiting an excessive response to LPS. Moreover, by this effect, the extracts also exert a protective, anti-inflammatory activity in the absence of specific inflammatory signals.

### 3.3. Effect of Chestnut Extracts on mRNA Levels of TLR4 and CD14 in LPS-Stimulated BV-2 Microglial Cells

**Figure 3:**
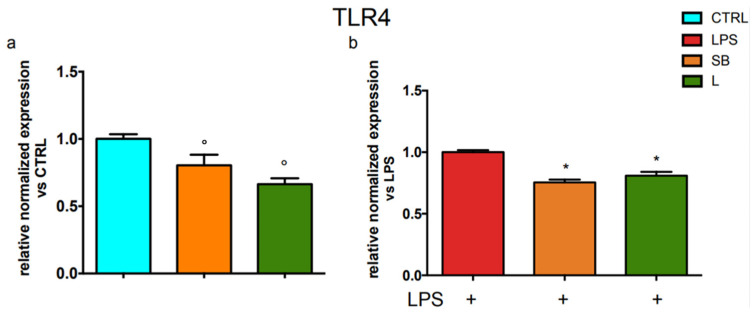
RT-PCR analysis of TLR4 gene expression in BV-2 cells treated with chestnut extracts. BV-2 cells were treated with spiny bur or leaf extracts (0.5 mg/mL) for 3 h, then stimulated (or not) with LPS (0.5 µg/mL) for a total of 24 h. At the end of incubation, RNA was extracted from cells and samples were subjected to RT-PCR analysis using a specific primer for TLR4, as explained in the Materials and Methods section. The relative mRNA content of TLR4 was normalised to (**a**) untreated control and (**b**) untreated LPS control. Relative quantification is obtained as reported in the Materials and Methods section; results are expressed as means ± SEM of three independent experiments. Statistical analysis was performed by Fisher’s LSD test following one-way ANOVA. ° *p* < 0.05 significantly different from control cells; * *p* < 0.05 significantly different from LPS-treated cells.

To corroborate these results, we investigated whether the ability of SB and L to decrease the level of TLR4 on the surface of BV-2 cells may be due to a reduction of its transcriptional level. Therefore, the cells treated as previously reported were analysed by real-time PCR to evaluate TLR4 expression (Figure 3).

Figure 3a shows that the treatment with both SB and L significantly decreased the mRNA level of TLR4 by about 25% in unstimulated BV-2 cells. Figure 3b shows that when cells were treated with SB or L and stimulated with LPS, the TLR4 mRNA level was significantly reduced. These findings agree with the data obtained by flow cytometric analysis reported in Figure 2.

**Figure 4:**
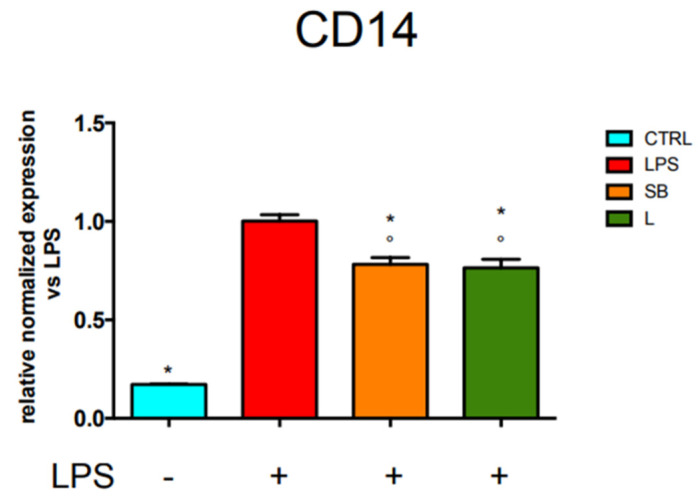
RT-PCR analysis of CD14 gene expression in BV-2 cells treated with chestnut leaf or spiny bur extracts. BV-2 cells were treated with spiny bur or leaf extracts (0.5 mg/mL) for 3 h, then stimulated (or not) with LPS (0.5 μg/mL) for a total of 24 h. At the end of incubation, RNA was extracted from cells and samples were subjected to RT-PCR analysis using a specific primer for CD14, as reported in the Materials and Methods section. The relative mRNA content of CD14 was normalised to untreated cells stimulated with LPS. Relative quantification is obtained as explained in the Materials and Methods section; results are expressed as means ± SEM of three independent experiments. Statistical analysis was performed by Fisher’s LSD test following one-way ANOVA. ° *p* < 0.05 significantly different from control cells; * *p* < 0.05 significantly different from LPS-treated cells.

The stimulation of mammalian cells with LPS occurs through an interaction with several proteins, among which the glycosylphosphatidylinositol-anchored protein CD14, which mediates the transfer of LPS to TLR4 and facilitates LPS recognition [28]. Due to the observed ability of the chestnut extracts to decrease TLR4 expression, we used PCR analysis to investigate the effect of the extracts on CD14 mRNA level. Results in Figure 4 show that CD14 mRNA level markedly and significantly increases in LPS-stimulated cells with respect to control cells but is significantly decreased when stimulated BV-2 cells were pre-treated with SB or L.

### 3.4. Effect of Chestnut Extracts on the NF-kB Signal Transduction Pathway in BV-2 Microglial Cells

**Figure 5:**
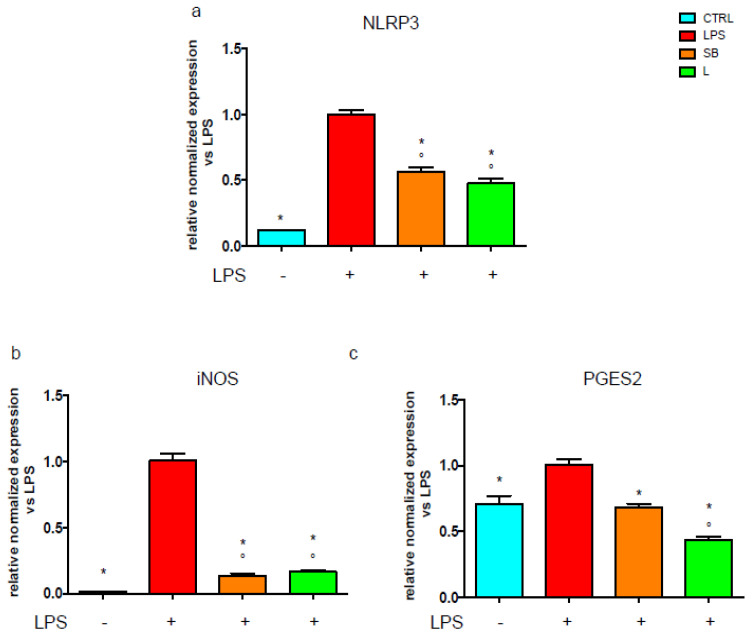
RT-PCR analysis of inflammatory markers gene expression in BV-2 cells treated with chestnut extracts. BV-2 cells were treated with spiny bur (SB) or leaf (L) extracts (0.5 mg/mL) for 3 h, then stimulated (or not) with 0.5 μg/mL LPS (red bars) for a total of 24 h. At the end of incubation, RNA was extracted, and samples were subjected to RT-PCR analysis using specific primers for (**a**) NLRP3, (**b**) iNOS, and (**c**) PGES2, as reported in the Materials and Methods section. The relative mRNA contents were normalised to LPS-treated cells. Relative quantification is obtained as described in the Materials and Methods section; results are expressed as means ± SEM of three independent experiments. Statistical analysis was performed by Fisher’s LSD test following one-way ANOVA. ° *p* < 0.05 significantly different from control cells; * *p* < 0.05 significantly different from LPS-treated cells.

In the present study, we have extended the analysis of inflammation markers linked to the NF-kB pathway to better elucidate the molecular mechanisms underpinned by the anti-inflammatory activity of the extracts (Figure 5).

As recently demonstrated, TLR4 is also involved in the activation of the NLRP3 inflammasome [29], which is a multimeric complex of many proteins, among which NLRP3 is the main one. Considering that NLRP3 expression is triggered in an NF-kB-dependent manner, we investigated the mRNA level of this protein by RT-PCR. Figure 5a shows that, upon LPS stimulation, BV-2 cells treated with SB or L exhibit a significantly lower level of NLRP3 mRNA.

Through the NF-kB pathway, LPS triggers the increased production of NO and PGE_2_, up-regulating the enzymes responsible for their synthesis, i.e., inducible Nitric Oxide Synthase (iNOS) and Prostaglandin E Synthase 2 (PGES2), respectively. With this in mind, we evaluated the effect of the extracts in LPS-activated BV-2 cells on iNOS and PGES2 expression. Both SB and L strongly and significantly down-regulate iNOS (Figure 5b) with respect to LPS-activated cells. Interestingly, cells treated with SB exhibit levels of PGES2 similar to unstimulated cells, while cells treated with L show even lower PGES2 mRNA content with respect to unstimulated cells (Figure 5c).

These findings indicate that SB and L reduce NO and PGE2 production by diminishing the expression levels of their encoding genes. Moreover, SB and L down-regulate the level of NLRP3 (Figure 5a).

### 3.5. Phytochemical Investigation of C. sativa Leaf Extracts

In this study, after being resuspended in water, *C. sativa* leaf extract (L) was subjected to liquid–liquid partition with solvents at different polarities (hexane, ethyl acetate and butanol) in order to obtain fractions enriched with different classes of compounds. Following this procedure, four fractions (Fr. Hex, Fr. EtOAc, Fr. But, and Fr. H_2_O) were obtained and tested in the bioassays.

**Figure 6:**
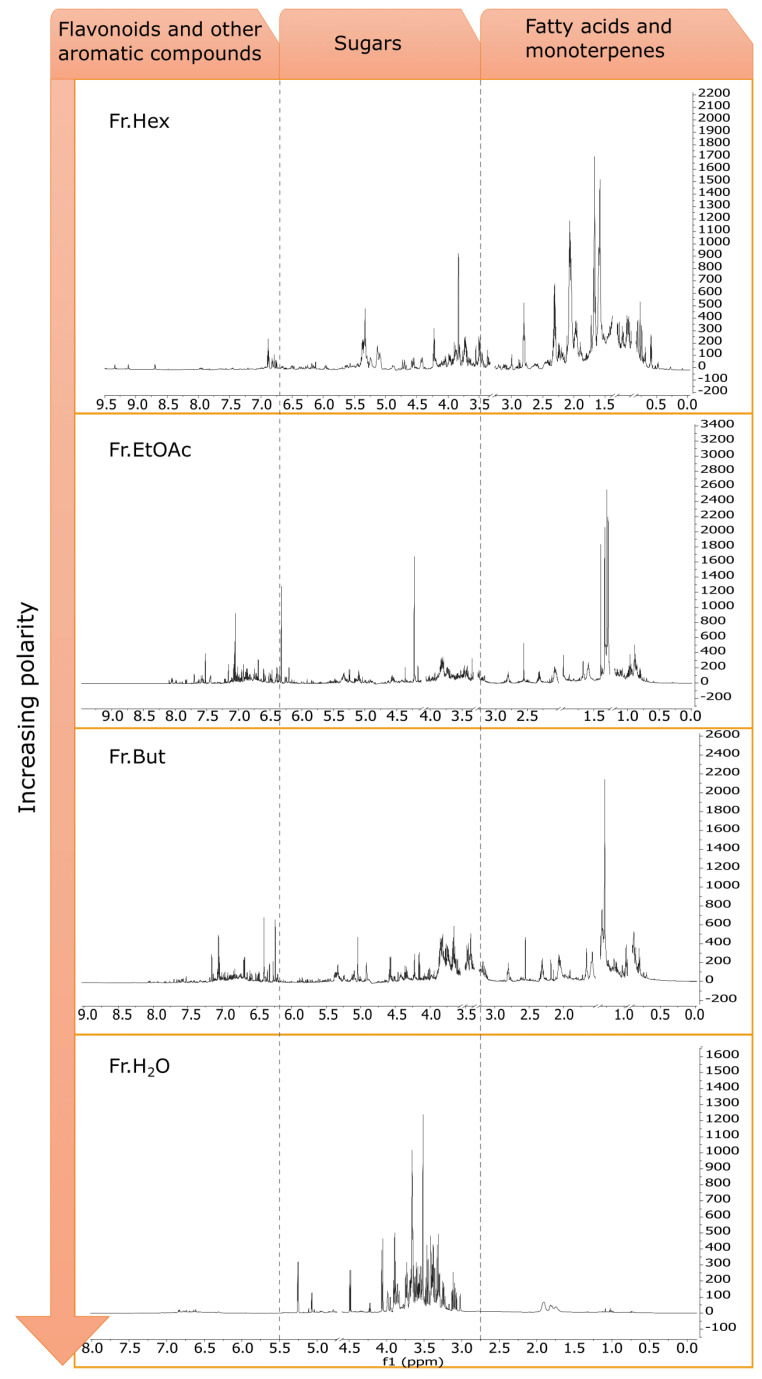
^1^H NMR profiling of the four fractions obtained by liquid/liquid partition of chestnut leaf extract (L). Spectral regions with the residual solvent signals were removed. Spectra of Fr. Hex, Fr. EtOAc and Fr. But were measured in CD_3_OD. The spectrum of Fr. H_2_O was acquired in a blend of CD_3_OD:D_2_O (1:1).

To attempt the identification of the most abundant metabolites, an overview of the phytochemical profiles of the four fractions was acquired by ^1^H NMR fingerprinting (Figure 6) and subsequently, further chromatographic procedures were carried out on Fr. EtOAc.

Fr. Hex ^1^H NMR profile is the richest in signals between δ 0.5–3 since this fraction contains the most apolar constituents. In particular, the presence of unsaturated fatty acids is particularly evident in the spectrum of this fraction. This is highlighted by the signals at δ 5.3 and 2.8, which are ascribable to olefinic protons, and to the protons bound to the bis-allylic carbons, respectively. Moreover, in the spectrum, the diagnostic signals of the protons in α-position to the carboxyl group (a doublet of doublets at δ 2.2), the protons bound to the allylic carbons (δ 2.0–2.1), the signal of the methylene protons at δ 1.2–1.4, and the protons of the terminal methyl group (δ 0.8–0.9) are visible [30].

**Figure 7:**
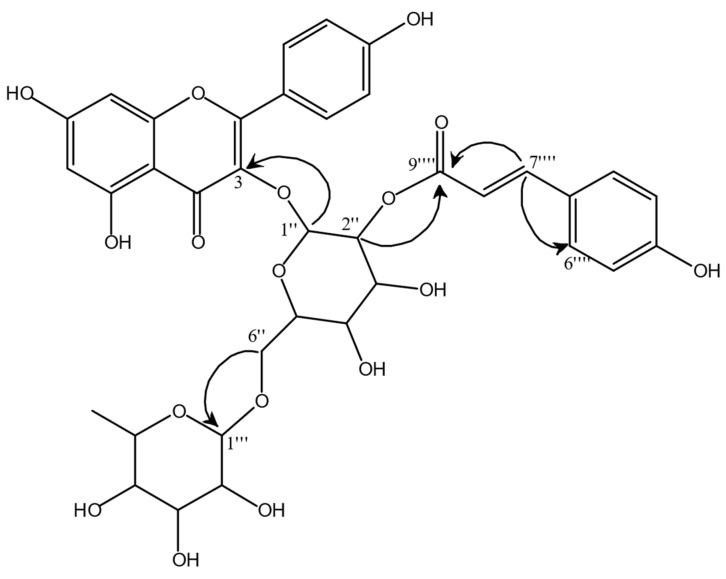
Kaempferol 3-rhamnosyl (1-6)(2″-trans-p-coumaroyl)hexoside isolated in fraction FR8. The arrows highlight the most important C-H correlation found through the HMBC experiment, allowing us to draw the connections between the different molecular moieties.

Also, the ^1^H NMR profile of Fr. EtOAc shows the presence of unsaturated fatty acids, even though at a lower concentration than Fr. Hex. On the other hand, Fr. EtOAc spectrum shows numerous signals in the region between δ 6.00 and 8, typically related to aromatic compounds. In our previous work [13], specific flavonoids in this fraction were already reported, namely astragalin, isorhamnetin glucoside, and myricitrin, which were characterised from the subfraction FR6 obtained by Fr. EtOAc column chromatography. Performing the same chromatography procedure, the subfraction FR8 yielded another flavonoid with a more complex structure, identified as kaempferol 3-rhamnosyl (1-6)(2″-trans-p-coumaroyl)hexoside. The most important C-H correlations for the structure elucidation, found through HMBC (Heteronuclear Multiple Bond Correlation) experiments, are highlighted in Figure 7.

In particular, the position of the glycosidic bond between kaempferol moiety and glucose moiety was clarified by the HMBC correlation between the anomeric proton of the glucoside at δ 5.55 and the carbon at δ 133.28. The rhamnose moiety results are linked to the position 6″ of glucose. In fact, both protons in 6″ of the glucoside (δ 3.86 and 3.42) give HMBC correlation with the carbon at δ 100.88, in turn, correlated in HSQC (Heteronuclear Single Quantum Coherence) to the anomeric proton of rhamnose (δ 4.53). Finally, the HMBC experiment also reveals the position of the p-coumaroyl moiety within the molecule through the correlation of the proton 2″ of the glucoside moiety (δ 5.00) with the carbonyl group (δ 166.65). Moreover, the coupling constant of the olefinic protons at δ 7.68 and 6.36 was 15.98 Hz, typical of the trans isomer. This molecule was identified as the main constituent of FR8, as also confirmed by its UHPLC-MS analysis, showing a prominent chromatographic peak absorbing at 254 and 365 nm, which MS pattern depicts a parent ion [M+H]^+^ at m/z 741.27. To the best of our knowledge, kaempferol 3-rhamnosyl (1-6)(2″-trans-p-coumaroyl)hexoside is a new molecule, characterised here for the first time. Structural analogues of this molecule, having the coumaroyl moiety bound to the carbon 4″ instead of 2″, were isolated from *Strychnos variabilis* [31,32].

In FR8, a less intense peak was also detected by UHPLC-MS analysis and was tentatively identified as tiliroside since it shows two main ions, namely [M+H]^+^ at m/z 595.26 and [M+Na]^+^ at m/z 617.07. Tiliroside is another well-known flavonoid acylated with p-coumaric acid moiety, and it was previously found in *C. sativa* burs by Esposito et al. [33].

Fr. But share some common compounds with Fr. EtOAc. In fact, signals ascribable to isorhamnetin glucoside and myricitrin are still evident in the ^1^H NMR profile of this fraction, namely the doublet at δ 7.70 and the doublet of doublets at δ 7.58 for isorhamnetin moiety, and a singlet at δ 7.52 for myricitrin. However, these flavonoids are less concentrated than in Fr. EtOAc. On the other hand, Fr. But contains a tannin-like compound having two singlets at δ 6.40 (HSQC correlation at δ 106.48) and δ 6.23 (HSQC correlation at δ 107.12), likely ascribable to gallic acid moieties.

Fr. H_2_O yields the most polar compounds extracted from chestnut leaves, which are mainly primary metabolites. The most abundant compounds are sucrose, α-glucose, and β-glucose, identified from the diagnostic signals generated by their anomeric protons at δ 5.40 (doublet, J = 3.9 Hz), at δ 5.2 (doublet, J = 3.8 Hz) and δ 4.60 (doublet, J = 7.9 Hz), respectively. Quinic acid is also detected by the broad multiplets between δ 1.85 and 2.13. Traces of the signals ascribable to aromatic compounds are also present at low magnetic fields between δ 6.40–7.2.

### 3.6. Evaluation of the Anti-Inflammatory Activity of the Fractions Obtained from Liquid/Liquid Partition of Chestnut Leaf Extract

**Figure 8:**
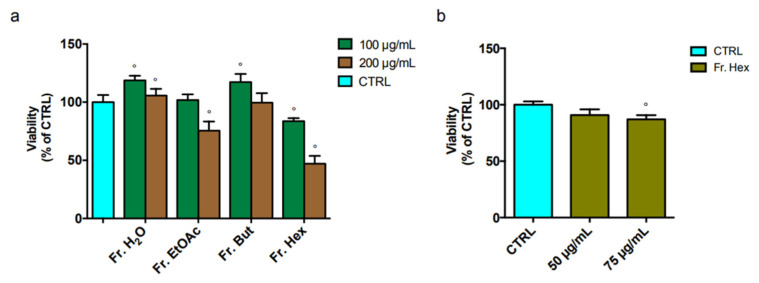
Cell viability of BV-2 treated with different concentrations of chestnut leaf extract fractions. BV-2 cells were treated for 3 h with (**a**) 100 or 200 μg/mL of different leaf-derived extract fractions or (**b**) 50 or 75 μg/mL of leaf-derived Fr. Hex (olive bars). Viability was evaluated by MTT assay, as described in the Materials and Methods section. Each bar represents the mean of n = 10 samples ± SEM. Statistical analysis was performed by Fisher’s LSD test following one-way ANOVA. ° *p* < 0.05 significantly different from control cells.

At first, the cytotoxicity of the different fractions was investigated by MTT assay. The viability of BV-2 cells after 24 h incubation with 100 or 200 μg/mL of leaf fractions was evaluated and reported in Figure 8a. Fr. EtOAc was cytotoxic only at 200 μg/mL, while Fr. H_2_O and Fr. But did not show cytotoxicity. Fr. Hex showed the highest cytotoxicity, significantly reducing cell viability compared to untreated cells at both concentrations. We then tested two lower concentrations of Fr. Hex (Figure 8b) and observed that only 75 µg/mL exerts a slight cytotoxic effect. Therefore, the concentration of 50 μg/mL was chosen for the following experiments.

**Figure 9:**
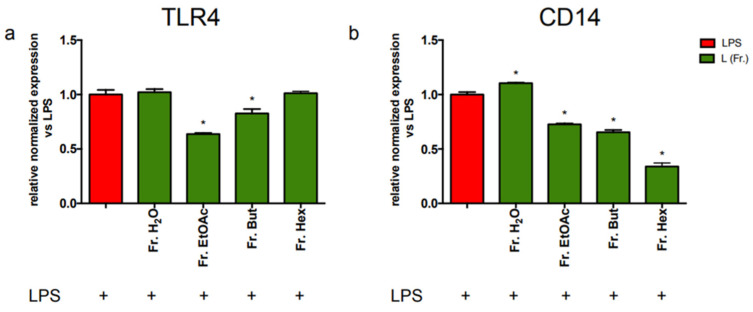
RT-PCR analysis of TLR4 and CD14 gene expression in BV-2 cells treated with different chestnut leaf extract fractions. BV-2 cells were treated for 3 h with 50 μg/mL of fractions at different polarity (L Fr.), then stimulated with 0.5 μg/mL LPS for a total of 24 h. At the end of incubation, RNA was extracted from cells and samples were subjected to RT-PCR analysis using a specific primer for TLR4 (**a**) and CD14 (**b**), as reported in the Materials and Methods section. The relative mRNA contents of TLR4 and CD14 were normalised to LPS-treated cells (red bars). Relative quantification is obtained as described in the Materials and Methods section, and results are expressed as means ± SEM of three independent experiments. Statistical analysis was performed by Fisher’s LSD test following one-way ANOVA. * *p* < 0.05 significantly different from LPS-treated cells.

Previously, we observed that leaf extract was able to reduce the expression of both TLR4 and CD14 in BV-2 cells (Figure 3 and Figure 4). To clarify which fraction may be the most relevant in mediating these effects, BV-2 cells were treated with the four different leaf-derived extract fractions before measuring, and the mRNA level of TLR4 and CD14 was investigated (Figure 9).

Results in Figure 9a show that Fr. EtOAc can decrease TLR4 expression by about 40% and Fr. But by about 20%, whereas Fr. H_2_O and Fr. Hex are ineffective. Figure 9b shows that both Fr. EtOAc and Fr. But decrease CD14 mRNA by about 30%, but in this case, Fr. Hex is the most effective in reducing CD14 expression (about 75%).

**Figure 10:**
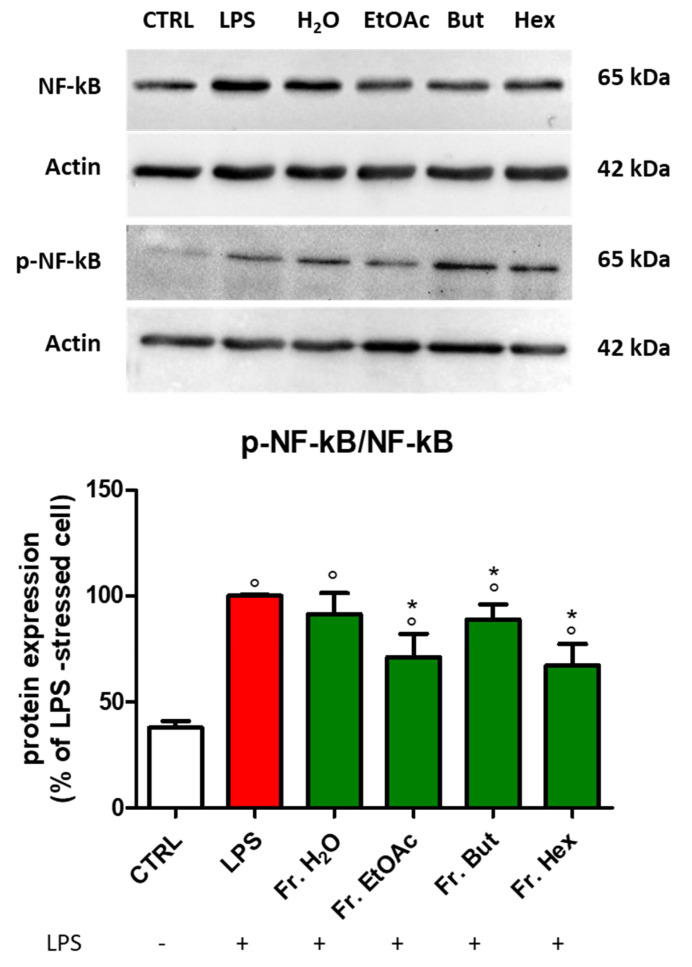
NF-kB and p-NF-kB protein expression in LPS-stimulated BV-2 cells treated with different chestnut leaf extract fractions. BV-2 cells were treated for 3 h with 50 μg/mL of fractions at different polarities obtained from L, then stimulated (or not) with 0.5 μg/mL LPS for 24 h. After cell lysis, the extracted proteins were separated by SDS-PAGE, transferred to a nitrocellulose membrane, and immunoassayed using anti-NF-kB or anti-p-NF-kB and anti-actin antibodies, as reported in the Materials and Methods section. Immunoblots are representative of three independent experiments; bars represent the densitometric analysis of immunoblotting results. Normalised expression levels were calculated relative to LPS-treated cells (red bars). Results are expressed as means ± SEM. Statistical analysis was performed by Fisher’s LSD test following one-way ANOVA. ° *p* < 0.05 significantly different from control cells; * *p* < 0.05 significantly different from LPS-treated cells.

With the aim of understanding which class of compounds could be responsible for the observed effects on the NF-kB transduction pathway, BV-2 cells previously treated with the four leaf-derived fractions were stimulated with LPS, and then protein levels of both NF-kB and its phosphorylated form were evaluated by Western blot analysis (Figure 10). As expected, LPS significantly increased both NF-kB and phospho-NF-kB levels with respect to control cells (Figure 10). The p-NF-kB/NF-kB ratio was particularly decreased in the presence of Fr. EtOAc and Fr. Hex.

**Figure 11:**
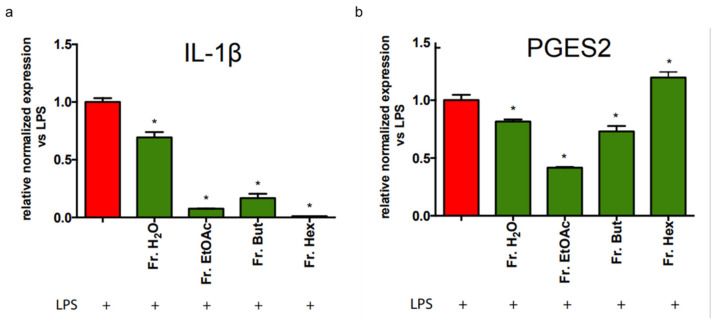
RT-PCR analysis of IL-1β and PGES2 gene expression in BV-2 cells treated with different chestnut leaf extract fractions. BV-2 cells were treated for 3 h with 50 μg/mL of different leaf-derived extract fractions, then stimulated with 0.5 μg/mL LPS for a total of 24 h. At the end of incubation, RNA was extracted from cells and samples were subjected to RT-PCR analysis using a specific primer for IL-1β (**a**) and PGES2 (**b**), as reported in the Materials and Methods section. The relative mRNA contents were normalised to LPS-treated cells (red bars). Relative quantification is obtained as described in the Materials and Methods section; results are expressed as means ± SEM of three independent experiments. Statistical analysis was performed by Fisher’s LSD test following one-way ANOVA. * *p* < 0.05 significantly different from LPS-treated cells.

We also evaluated the effect of the different leaf extract fractions on the inflammatory markers linked to the NF-kB pathway. To this purpose, BV-2 cells were stressed by LPS, and the expression of IL-1β and PGES2 was analysed by RT-PCR. As reported in Figure 11a, Fr. EtOAc, Fr. But and Fr. Hex strongly inhibited IL-1β gene expression, whereas Fr. H_2_O caused an expression reduction of about 25%. Figure 11b shows that the mRNA level of PGES2 was strongly decreased in the presence of Fr. EtOAc (about 60%) and was diminished by about 20% by Fr. H_2_O and Fr. But. Fr. Hex did not affect PGES2 mRNA level.

**Figure 12:**
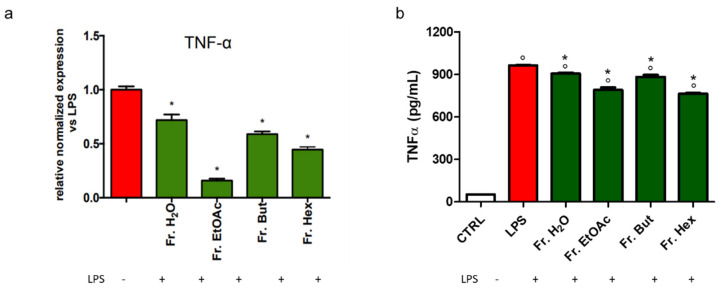
Gene expression and protein content of TNF-α in BV-2 cells treated with different chestnut leaf extract fractions. BV-2 cells were treated for 3 h with 50 μg/mL of different leaf-derived extract fractions, then stimulated with 0.5 μg/mL LPS for a total of 24 h. (**a**) At the end of incubation, RNA was extracted from cells and samples were subjected to RT-PCR analysis using a specific primer for TNF-α, as explained in the Materials and Methods section. The relative mRNA contents were normalised to LPS-treated cells (red bars). Relative quantification is obtained as described in the Materials and Methods section (**b**) Cellular content of TNF-α was quantified by ELISA assay as described in the Materials and Methods section. Results are expressed as means ± SEM of three independent experiments. Statistical analysis was performed by Fisher’s LSD test following one-way ANOVA. ° *p* < 0.05 significantly different from control cells; * *p* < 0.05 significantly different from LPS-treated cells.

Another LPS-induced inflammatory cytokine linked to the pathway of NF-kB is the tumour necrosis factor α (TNF-α); thus, this cytokine was measured both at the protein and gene expression levels. Figure 12a indicates that the stimulatory effect of LPS on TNF-α mRNA transcription was significantly inhibited in BV-2 cells pre-treated with the different leaf extract fractions, among which Fr. EtOAc and Fr. Hex showed the best activity. The LPS-induced TNF-α cellular content (Figure 12b) was significantly reduced in the cells pre-treated with the different leaf fractions, although at a lesser extent than that observed for the transcriptional level, and again Fr. EtOAc and Fr. Hex were the most effective.

**Figure 13:**
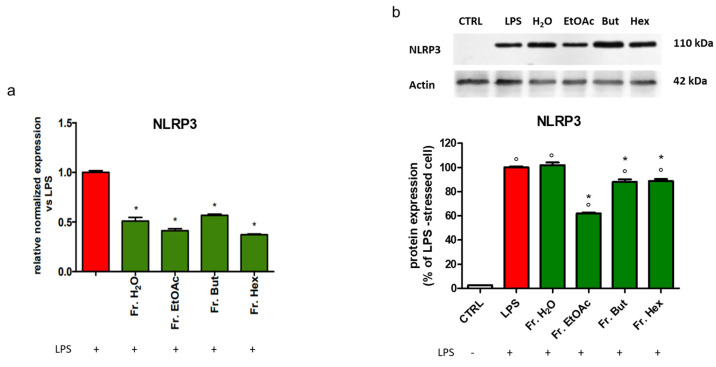
Gene expression and protein level of NLRP3 in BV-2 cells treated with different chestnut leaf extract fractions. BV-2 cells were treated for 3 h with 50 μg/mL of different leaf-derived extract fractions, then stimulated with 0.5 μg/mL LPS for a total of 24 h. (**a**) At the end of incubation, RNA was extracted from cells and samples were subjected to RT-PCR analysis using a specific primer for NLRP3, as described in the Materials and Methods section. The relative mRNA contents were normalised to LPS-treated cells (red bars). Relative quantification is obtained as described in the Materials and Methods section, and results are expressed as means ± SEM of three independent experiments. (**b**) After cell lysis, the extracted proteins were separated by SDS-PAGE, transferred to a nitrocellulose membrane, and immunoassayed using anti-NLRP3 and anti-actin antibodies, as explained in the Materials and Methods section. Immunoblot is representative of three independent experiments; bars represent the densitometric analysis of immunoblotting results. Normalised expression levels were calculated relative to LPS-treated cells (red bars). Statistical analysis was performed by Fisher’s LSD test following one-way ANOVA. ° *p* < 0.05 significantly different from control cells; * *p* < 0.05 significantly different from LPS-treated cells.

In addition, the expression of NLRP3 inflammasome is positively modulated by NF-kB and linked to the TLR4 pathway. Figure 13a shows that the mRNA level of NLRP3 was significantly reduced in LPS-stimulated BV-2 cells pre-treated with the different leaf extract fractions. Of note, the higher activity in decreasing the LPS-induced NLRP3 protein expression was exhibited by the EtOAc fraction (Figure 13b), whereas Fr. But and Fr. Hex were less effective.

**Figure 14:**
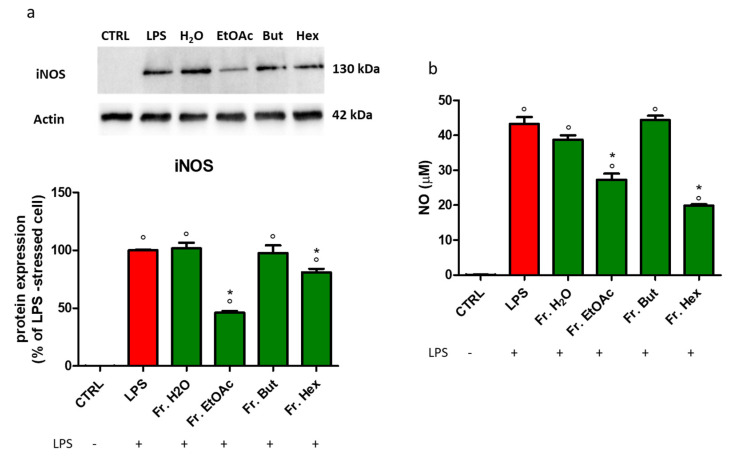
iNOS protein expression and NO level in BV-2 cells treated with different chestnut leaf extract fractions. BV-2 cells were treated for 3 h with 50 μg/mL of different leaf-derived extract fractions (L Fr.), then stimulated with 0.5 μg/mL LPS for a total of 24 h. (**a**) After cell lysis, the extracted proteins were separated by SDS-PAGE, transferred to a nitrocellulose membrane, and immunoassayed using anti-iNOS and anti-actin antibodies, as reported in the Materials and Methods section. Immunoblot is representative of three independent experiments; bars represent the densitometric analysis of immunoblotting results. Normalised expression levels were calculated relative to LPS-treated cells (red bars) (**b**) NO level was quantified by Griess assay as described in the Materials and Methods section. Results are expressed as means ± SEM of three independent experiments. Statistical analysis was performed by Fisher’s LSD test following one-way ANOVA. ° *p* < 0.05 significantly different from control cells; * *p* < 0.05 significantly different from LPS-treated cells.

The induced isoform of NOS (iNOS) is a stress-induced enzyme that is expressed only after cell stimulation, typically by pro-inflammatory cytokines and/or bacterial LPS. After iNOS induction, a significant amount of the free radical NO is generated. Thus, the iNOS protein expression and the quantification of NO release were measured. As reported in Figure 14a, BV-2 treatment with EtOAc fraction caused a decrease of about 60% in LPS-induced iNOS cellular content. Cells treated with Fr. Hex diminished iNOS content by about 20%, whereas the cell treatment with Fr. H_2_O and Fr. But did not exert any effect. The LPS-induced NO release was significantly reduced only in the presence of Fr. Hex and Fr. EtOAc, according to the results shown in Figure 14a.

## 4. Discussion

In our previous study [13], we evidenced cytoprotective and anti-inflammatory activities played by extracts derived from leaves and spiny burs harvested from different *Castanea sativa* cultivars in LPS-stimulated BV-2 cells. The aim of the present study was to clarify the molecular mechanism underlying the anti-inflammatory effect of extracts obtained from leaves and spiny burs using the same neuroinflammatory model. LPS-activated microglial cells have been proposed as a suitable cellular model to test the potential efficacy of compounds in counteracting neuroinflammatory disorders [34], and the role of activated microglia in central nervous system inflammation is well established [35]. Stimulation of microglia with Toll-like receptor (TLR) agonists, including LPS for TLR4, leads to the accumulation of cytotoxic compounds such as ROS and pro-inflammatory cytokines, including IL-1β, IL-6, TNF-α as well as nitric oxide (NO) and chemokines [36]. Excessive activation of TLR4 on microglia may lead to damage and neuronal loss. Therefore, the modulation of TLR4 signalling could be a potential therapeutic target in neurodegenerative diseases.

In particular, the extracts studied in this research project, SB and L, were obtained by pooling leaves or spiny burs collected from different chestnut trees, with the aim of getting general results that can be associated with any extract obtained from these matrices. This is especially relevant when using waste products whose exact origin is not always easy to identify, as in the case of a circular economy perspective.

Our results show that both extracts did not directly influence the binding of LPS to TLR4, as demonstrated by cytofluorimetric analysis with FITC-LPS, but markedly reduced the level of TLR4 on BV-2 cell membrane through the downregulation of TLR4 expression. Therefore, we can suggest that the treatment with chestnut extracts reduced the response of BV-2 cells to LPS, at least in part, by decreasing the number of membrane receptors able to interact with LPS, thus partially blocking its inflammatory effect. According to this observation, the mRNA level of the protein CD14, which cooperates in the activation of TLR4 by LPS, is significantly decreased when LPS-stimulated BV-2 cells were pre-treated with chestnut extracts. We cannot exclude LPS insertion into the lipid bilayer, as reported by Wurfel and Wright [37]; however, we focused on the effect of chestnut by-product extracts on the LPS signal transduction mediated by TLR4.

Data in the literature report similar effects in LPS-stimulated BV-2 cells pre-treated with isorhamnetin [38] or kaempferol [39], but in both cases, the effect of the two flavonoids was evident only in stimulated cells. The two well-known antioxidants, isorhamnetin [40] and kaempferol [41], are flavonoids generally found in fruits and leaves of various plants, but their presence has also been reported among the metabolites found in *Castanea sativa* (“Marrone di Roccadaspide”) [11] and in *Castanea sativa* from the chestnut grove of Granaglione, described in the present study. Therefore, the ability of leaf and spiny bur extracts to block the activity of TLR4 receptors linked to the neuroinflammation pathway may be ascribed, at least in part, to the presence of these two flavonoids.

LPS binding to TLR4 triggers many signal transduction pathways that, in turn, lead to the stimulation of the transcription factor NF-kB, through which a series of pro-inflammatory genes are expressed, such as those encoding for iNOS, COX-2, PGES2 enzymes, and many inflammatory cytokines are produced [28,42], such as IL-1β, TNF-α, the eicosanoid PGE2, as well as the activation of NLRP3 inflammasome.

In our previous paper, we demonstrated that BV-2 cells treated with leaf and spiny bur extracts exhibited a lower expression of the inflammatory mediators IL-1β and TNF-α, together with a reduced expression of NF-kB. The present study demonstrates that SB and L strongly suppress iNOS expression and, to a lesser extent, PGES2 expression in LPS-stimulated BV-2 cells. iNOS and cyclooxygenase-2 (COX-2) belong to a family of primary inflammatory genes transcribed in response to activated microglia. Evidence suggests that iNOS is responsible for the main NO production after inflammatory insult in the brain, and it is generally accepted that excessive production of NO can contribute to neurodegeneration [39].

PGE2, a major eicosanoid found in many inflammatory conditions, is converted from PGH2 by PGES2 and PGH_2_ is synthesised from arachidonic acid by COX-2. Studies using inhibitors of iNOS and COX-2 expression have demonstrated significant neuroprotection in several models of neurodegeneration and inflammation [43,44].

Many data reported in the literature corroborate the results present in this study. Cerulli and co-workers showed that the pre-treatment of THP1 and THP-1-XBLUE-MD2-CD14 cells with extracts of *Castanea sativa* shells [45] or leaves and burs [11] inhibited both NF-kB activity and NO production after LPS stimulation. The suppression of the excessive NO production and iNOS expression was also reported by Kang [46] in BV-2 cells pre-treated with chestnut (*Castanea crenata*) peel extracts and stimulated with LPS. Other authors obtained the same results in LPS-stimulated microglia cells treated with isolated flavonoids also found in *Castanea*, such as kaempferol [39], isorhamnetin [38], or astragalin [47].

It has recently been established that TLR4 is also involved in the activation of NLRP3 inflammasome, a multimeric complex which ultimately serves to induce autoproteolysis and activation of caspase-1 [20]. Activation of TLR4 serves as a priming signal promoting the expression of NLRP3, whereas a second stimulus triggers the assembly of the fully active inflammasome. Since the expression of NLRP3 is positively modulated in an NF-kB-dependent manner, both signalling pathways triggered by TLR4 can, in principle, be involved. Our results show that both TLR4 and NF-kB activation is reduced in cells pre-treated with chestnut extracts. Therefore, the significant reduction in the mRNA level of NLRP3 observed in LPS-stimulated BV-2 cells fit with the inhibition of these synergic mechanisms.

By means of liquid chromatography coupled to mass-spectrometry, Cerulli and co-workers [11] analysed the MeOH extract of “Marrone di Roccadaspide” burs and leaves, identifying glycosylated flavonols such as quercetin, isorhamnetin and kaempferol derivatives. In our previous work [13], the presence of specific flavonoids in Fr. EtOAc of leaf extract from *Castanea sativa*, namely astragalin, isorhamnetin glucoside, and myricitrin was reported, and the presence of these flavonoids has been confirmed in the extracts analysed in the present study. Results described here also reveal the presence, in the same fraction, i.e., Fr. EtOAc, of a new flavonoid, namely kaempferol 3-rhamnosyl (1-6)(2″-trans-p-coumaroyl)hexoside.

Aiming to clarify the contribution of different classes of molecules contained in L to the observed anti-inflammatory effect, this raw extract was subjected to liquid/liquid partition with solvents at different polarities, chosen among those commonly employed for extraction from vegetal sources. As a first step, we decided to subject only the leaf extract to this treatment since, although the two extracts were both found to be effective in counteracting neuroinflammation, the extract obtained from the leaves demonstrated a slightly superior effect. Moreover, it has been reported that the recovery of the highest quantity of bioactive compounds (mainly flavonoids and tannins) is especially obtained from leaves, followed by burs and shells [48]. The fractions obtained by liquid/liquid partition of leaf extract were analysed by ^1^H NMR fingerprinting, showing the presence of several compounds such as fatty acids, flavonoids, coumaroyl flavonoids and tannins.

Fr. EtOAc, Fr. But and Fr. Hex were the most effective in reducing the TLR4 and CD14 gene expression. Since activation of TLR4 leads to phosphorylation and activation of NF-kB, we measured the LPS-induced BV-2 cellular content of both NF-kB and its phosphorylated form, showing that they were significantly reduced in the presence of Fr. EtOAc and Fr. Hex, whereas Fr. But was effective only on the level of p-NF-kB. Consequently, we investigated the gene expression of some inflammatory markers linked to the NF-kB pathway in LPS-stimulated BV-2 cells, such as IL-1β, PGES2, TNF-α and NLRP3 and found that all the fractions tested were able to significantly decrease their mRNA content, although at different extent, confirming the best effectiveness of Fr. EtOAc. To corroborate our findings, we also measured the LPS-stimulated BV-2 cellular content of TNF-α and NLRP3, obtaining results in agreement with the previous ones. Fr. EtOAc and Fr. Hex were also the most effective in reducing iNOS protein levels and the consequent release of NO in the culture medium.

Regarding Fr. EtOAc and Fr. But and according to data in the literature, it is likely that these effects are due to the presence of specific flavonoids in these fractions, such as isorhamnetin glucoside, astragalin, myricitrin, kaempferol 3-rhamnosyl (1-6)(2″-trans-p-coumaroyl)hexoside and tiliroside.

It has been reported that isorhamnetin suppresses LPS-mediated inflammatory action in BV-2 cells by downregulating the NF-kB signalling pathway, antagonising TLR4 and decreasing ROS level [38]. Isorhamnetin could be considered a potential agent to prevent Alzheimer’s and other neurodegenerative diseases [49].

Astragalin reduced LPS-induced synthesis of NO, iNOS, IL-1β and TNF-α in the microglia and hippocampus of mice [47], and myricitrin diminished the amount of pro-inflammatory factors and suppressed the expression of COX-2 and iNOS [50].

In an extensive paper, Park et al. [39] analysed the anti-inflammatory properties of kaempferol and its mechanisms of action in microglial BV-2 cells previously stimulated by LPS. By means of the down-regulation of TLR4, NF-kB, p38 MAPK, JNK and AKT, kaempferol was able to reduce inflammatory mediators, and consequently, it could play a role in the treatment of neuroinflammatory diseases. Thus, the presence of kaempferol 3-rhamnosyl (1-6)(2″-trans-p-coumaroyl)hexoside in the L extract could add value to chestnut by-products as a source of bioactive kaempferol. Moreover, tiliroside is endowed with numerous biological activities, i.e., anti-inflammatory, hepatoprotective, antidiabetic, and neuroprotective [51] and, notably, has been reported to exert anti-neuroinflammatory activities on BV-2 microglia [52].

Fr. Hex is the most efficient in reducing CD14 and IL-1β gene expression, showing a significant effect on the reduction of the phosphorylated form of NF-kB protein expression and the best effectiveness in reducing the NO amount induced by LPS in BV-2 cells. The ^1^HNMR spectrum of this fraction shows the presence of unsaturated fatty acids, which could be responsible for the observed effects. In fact, n-3 polyunsaturated fatty acids can decrease the production of pro-inflammatory mediators, such as TNF-α, and alter the signalling pathways that regulate gene expression, such as NF-kB activity [53,54], helping to counteract endothelial dysfunction.

In conclusion, the present study demonstrates that extracts from chestnut leaves or spiny burs exert potent anti-inflammatory effects on BV-2 microglial cells stimulated with LPS, mainly associated with their ability to reduce TLR4 expression. Leaf extract became an interesting source of bioactive compounds such as flavonoids, among which a new molecule was identified.

The main challenge concerning the assessment of the clinical efficacy of natural anti-inflammatory products is the bioavailability of natural bioactive molecules. In fact, they must overcome the impediment posed by the blood-brain barrier to act in the brain. Recently, an artificial membrane permeability assay (PAMPA-BBB) was adopted to assess the blood-brain barrier penetration of pharmacologically active natural products and plant extracts. The study by Sanchez-Martinez et al. [55] reported the PAMPA-BBB potential penetrability of 113 different compounds extracted from different plant matrices, among which isorhamnetin, kaempferol and quinic acid (present also in our chestnut extracts) resulted able to cross the blood–brain barrier potential, provided that the molecular structure does not contain sugars moieties. Other factors are responsible for the poor availability of flavonoids in vivo, such as their metabolic and microbial transformation, as reported in [56,57].

Taking into consideration the above-mentioned limitations, additional studies are needed to further investigate the properties of chestnut extracts both in cell models and in vivo. Nevertheless, the results of this study encourage the recycling and valorisation of *Castanea* by-products from a circular economy perspective: supplying bioactive, neuroprotective compounds and decreasing the environmental impact linked to chestnut grove waste.

## Figures and Tables

**Table 1:** Primer used for Real-time qPCR.

Target	Sequence (5′–3′)	ID RefSeq
GAPDH *	Forward ACCACAGTCCATGCCATCAC	NM_008084
	Reverse TCCACCACCCTGTTGCTGTA	
TLR4	Forward GATCAGAAACTCAGCAAAGTC	NM_021297
	Reverse TGTTTCAATTTCACACCTGG	
CD14	Forward GCC AAA TTG GTC GAA CAA GC	NM_009841
	Reverse CCA TGG TCG GTA GAT TCT GAA AGT	
NLRP3	Forward GATGCTGGAATTAGACAACTG	NM_145827
	Reverse GTACATTTCACCCAACTGTAG	
PGES2	Forward GAAGGACTGAGATCAAATTCTC	NM_008964
	Reverse ATGACAGAGGAGTCATTGAG	
IL-1β	Forward GTTCCCATTAGACAACTGCACTACAG	NM_008361
	Reverse GTCGTTGCTTGGTTCTCCTTGTA	
TNF-α	Forward CCCCAAAGGGATGAGAAGTTC	NM_013693
	Reverse CCTCCACTTGGTGGTTTGCT	
iNOS	Forward CCTCCTCCACCCTACCAAGT	NM_010927
	Reverse CACCCAAAGTGCTTCAGTCA	

* Reference gene.

## Data Availability

The original contributions presented in the study are included in the article. Further inquiries can be directed to the corresponding authors.
